# Value of Combined PET Imaging with [^18^F]FDG and [^68^Ga]Ga-PSMA-11 in mCRPC Patients with Worsening Disease during [^177^Lu]Lu-PSMA-617 RLT

**DOI:** 10.3390/cancers13164134

**Published:** 2021-08-17

**Authors:** Fadi Khreish, Kalle Ribbat, Mark Bartholomä, Stephan Maus, Tobias Stemler, Ina Hierlmeier, Johannes Linxweiler, Mathias Schreckenberger, Samer Ezziddin, Florian Rosar

**Affiliations:** 1 Department of Nuclear Medicine, Saarland University, 66421 Homburg, Germany; s8karibb@stud.uni-saarland.de (K.R.); mark.bartholomae@uks.eu (M.B.); stephan.maus@uks.eu (S.M.); tobias.stemler@uks.eu (T.S.); inamaria.hierlmeier@uks.eu (I.H.); samer.ezziddin@uks.eu (S.E.); florian.rosar@uks.eu (F.R.); 2 Department of Urology, Saarland University, 66421 Homburg, Germany; johannes.linxweiler@uks.eu; 3 Department of Nuclear Medicine, Johannes Gutenberg-University, 55131 Mainz, Germany; mathias.schreckenberger@unimedizin-mainz.de

**Keywords:** radioligand therapy, PSMA, FDG, PET/CT, mismatch, metastatic castration-resistant prostate cancer

## Abstract

**Simple Summary:**

Prostate-specific membrane antigen (PSMA) is regularly overexpressed in prostate cancer cells. Radioligand therapy (RLT) targeting PSMA has shown impressive results in recent years in treatment of metastatic castration-resistant prostate cancer (mCRPC). In some patients, however, the disease worsens during PSMA-RLT. A proportion of these patients develop a type of metastasis that has intense glucose metabolism but missing or low PSMA expression; these metastases are referred to as ‘mismatch metastases’. We found that combined PET imaging using the radiolabeled glucose analog [^18^F]FDG and the PSMA radioligand [^68^Ga]Ga-PSMA-11 is essential to identify patients with mismatch findings, which are associated with significantly worse outcome, especially if the mismatch is located in the liver or of high volume. Consequently, additional treatments or change to another treatment modality appears necessary.

**Abstract:**

Despite the promising results of prostate-specific membrane antigen (PSMA)-targeted radioligand therapy (RLT) in metastatic castration-resistant prostate cancer (mCRPC), some patients show worsening disease during PSMA-RLT. We investigated the value of combined [^18^F]FDG and [^68^Ga]Ga-PSMA-11 PET imaging in this setting. In *n* = 29 mCRPC patients with worsening disease after a median of four cycles of [^177^Lu]Lu-PSMA-617 RLT, combined [^18^F]FDG and [^68^Ga]Ga-PSMA-11 PET imaging was performed to detect [^18^F]FDG-avid lesions with low or no PSMA expression (mismatch lesions). To evaluate prognostic implication of mismatch, survival analyses regarding presence, location, and [^18^F]FDG PET-derived parameters such as SUV_max_, metabolic tumor volume (MTV_m_), and total lesion glycolysis (TLG_m_) of mismatch findings were performed. Seventeen patients (59%) showed at least one mismatch metastasis. From the time point of combined PET imaging, the median overall survival (OS) of patients with mismatch findings was significantly *(p* = 0.008) shorter than those without (3.3 vs. 6.1 mo). Patients with a high MTV_m_ revealed a significantly (*p* = 0.034) shorter OS of 2.6 mo than patients with low MTV_m_ (5.3 mo). Furthermore, patients with hepatic mismatch showed a significantly (*p* = 0.049) shorter OS than those without (2.9 vs. 5.3 mo). Difference in OS regarding SUV_max_ and TLG_m_ was not significant. In mCRPC patients with worsening disease during PSMA-RLT, combined [^18^F]FDG and [^68^Ga]Ga-PSMA-11 PET imaging is essential to identify mismatch findings, as these are associated with poor outcomes requiring a change in therapy management.

## 1. Background

Prostate cancer is one of the most common malignancies in men, with over 1,200,000 new cases and approximately 359,000 deaths worldwide in 2018 [1]. A significant proportion of patients ultimately progresses to lethal setting of metastatic castration-resistant prostate cancer (mCRPC) [2,3]. Besides chemotherapy with taxanes (docetaxel and cabazitaxel) [4,5] and novel androgen axis drugs (NAAD) (e.g., abiraterone and enzalutamide) [6,7], radioligand therapy (RLT) targeting the prostate specific membrane antigen (PSMA) is an effective therapy option in patients with mCRPC. PSMA-RLT using [^177^Lu]Lu-PSMA-617 showed impressive results (with only moderate side effects) in various retrospective studies [8,9,10,11], in prospective phase II trials [12,13], and in a recently published phase III trial [14]. An adequate PSMA expression is essential for the success of PSMA-targeted radioligand therapy (PSMA-RLT), and is verified by PSMA-targeted positron emission tomography (PET)/computed tomography (CT) with radiolabeled PSMA ligands, such as [^68^Ga]Ga-PSMA-11, before and during therapy [15]. Despite the promising results of PSMA-RLT, some patients do not exhibit a sufficient response, and others with initially good responses experience a worsening disease in the course of PSMA-RLT [16,17]. Some of those patients with worsening disease develop lesions with missing or low PSMA expression. To detect those lesions, [^18^F]FDG PET/CT seems to be a suitable imaging method [18,19]. Although mostly prostate carcinoma cells have a low glucose metabolism due to energy gain by lipids and other energetic molecules, in advanced mCRPC the glucose metabolism is very heterogeneous and can be highly increased by shifting to aerobic glycolysis after numerous mutation events [20]. Such lesions with intense glucose metabolism on [^18^F]FDG PET/CT but missing or low PSMA expression, so-called ‘mismatch findings’, may be insufficiently affected by PSMA-RLT, and may necessitate a change in therapy management. However, little is known about the impact of the development and value of diagnosing mismatch findings during treatment.

The aim of this study was to investigate the use of combined [^18^F]FDG and [^68^Ga]Ga-PSMA-11 PET imaging in patients with worsening disease during PSMA-RLT, and the impact of mismatch findings on survival outcome.

## 2. Methods

### 2.1. Study Design

This retrospective study comprised mCRPC patients who were imaged by [^68^Ga]Ga-PSMA-11 and [^18^F]FDG PET (within about 4 weeks) at the time point of worsening disease under ongoing PSMA-RLT. Worsening disease was defined as biochemical progression with a prostate-specific antigen (PSA) increase of more than 25% according to PCWG3 guidelines [21], clinical progression, or radiological progression. To avoid a potential flare phenomenon, only patients with at least 2 cycles of [^177^Lu]Lu-PSMA-617 RLT were included in this study. Patients with secondary malignancies were excluded to avoid potential interference of image interpretation.

### 2.2. Patients and Ethics

Twenty-nine patients with mCRPC were included in this retrospective analysis. The patients received a median of 4 cycles (range: 2–10 cycles) of [^177^Lu]Lu-PSMA-617 RLT before performing combined [^18^F]FDG and [^68^Ga]Ga-PSMA-11 PET imaging. Detailed information about the patient characteristics is presented in Table 1. The mean time between both PET/CT scans was 9 ± 11 days (range: 1–36 days). PSMA-RLT was performed on a compassionate use basis under the German Pharmaceutical Act §13 (2b). All patients were treated within a prospective patient registry (REALITY Study, NCT04833517). Patients gave written consent after being informed in detail about the risks and potential side effects of this intervention. Patients consented additionally to publication of any resulting data in accordance with the Declaration of Helsinki. The study was approved by the local institutional review board (ethics committee permission number 140/17, 13 July 2017).

### 2.3. PET Acquisition and Analysis

For PET imaging, a mean activity of 119 ± 13 MBq [^68^Ga]Ga-PSMA-11 (range: 97–147 MBq) and 273 ± 22 MBq [^18^F]FDG (range: 215–311 MBq) was administered approximately 60 min and 90 min before the PET scan, respectively. All PET examinations were performed as PET/CT scans using an EANM accredited Biograph 40 mCT PET/CT scanner (Siemens Medical Solutions, Knoxville, TN, USA). The PET acquisition was performed from vertex to mid-femur with 3 min ([^68^Ga]Ga-PSMA-11) or 2 min ([^18^F]FDG) per bed position, covering a 21.4 cm extended field of view. CT data were acquired with a low-dose technique using an X-ray tube voltage of 120 keV and a modulation of the tube current by applying ‘CARE Dose4D’ with a maximal tube current of 30 mAs. The PET data sets were reconstructed using an iterative 3-dimensional ordered-subset expectation maximization (OSEM) algorithm (3 iterations; 24 subsets) with gaussian filtering and a slice thickness of 5 mm. Random correction, decay correction, scatter correction, and attenuation correction were applied. Both PET/CT scans were read simultaneously by experienced physicians searching for mismatch findings. Typical inflammatory changes on [^18^F]FDG PET/CT (e.g., pulmonary inflammatory changes, reactive-inflammatory lymph nodes) were not considered. A mismatch finding was defined as metastasis with remarkable [^18^F]FDG uptake and no or considerably less concordant [^68^Ga]Ga-PSMA-11 uptake based on visual analysis. Metastases showing adequate PSMA expression independent of the glucose metabolism ([^18^F]FDG uptake) were defined as non-mismatch findings. For each mismatch lesion, we measured maximum standardized uptake value (SUV_max_) and quantified the metabolic tumor volume (MTV) and total lesion glycolysis (TLG), which are established parameters in [^18^F]FDG PET/CT imaging [22,23]. All PET quantifications were performed using Syngo.via (Siemens Medical Solutions, Knoxville, TN, USA). MTV was calculated by precise segmentation using an individual threshold (TS) as described in detail by Schaefer et al. [24]. This algorithm was developed for precise PET-based volumetric analysis, and uses the SUV_mean_ of a 70% SUV_max_ isocontour of the respective tumor lesion and the background surrounding the tumor lesion to calculate an individual TS, which is used for auto-contouring the tumor volume. The required system-specific calibration parameter used in the algorithm was performed by in-house phantom measurements. TLG of each lesion was calculated as the product of the MTV and the respective SUV_mean_. Metabolic tumor volume of all mismatch lesions (MTV_m_) was defined as the sum of the MTV of all mismatch metastases. In analogy, total lesion glycolysis of all mismatch lesions (TLG_m_) was defined as the sum of the TLG of all mismatch metastases.

### 2.4. Statistical Analyses

Analysis of overall survival (OS) based on the Kaplan–Meier method was performed using Prism version 8 (GraphPad Software, San Diego, USA) and SPSS version 25 (SPSS Inc., Chicago, IL, USA). OS was defined as the interval from start of PSMA-RLT to the occurrence of any of the following: (1) death from any cause, (2) the last study visit, or (3) initiation of a different treatment (e.g., chemotherapy). The cut-off follow-up date was 30 April 2021. Further analysis of the OS was performed starting from the date of the [^18^F]FDG PET. The patients with mismatch metastases were dichotomized in groups by presence or absence of liver metastases, as well as regarding the median value of the [^18^F]FDG PET-derived parameters. The median values were used as cut-off because the sample size was too small to derive a reliable optimal cut-off. Cox proportional-hazards regression on univariate analysis (with metric variables) was performed between OS and [^18^F]FDG PET–derived parameters from the time point of diagnosis of mismatch lesions. Correlation between [^18^F]FDG PET-derived parameters and PSA serum levels was calculated using Spearman’s rank correlation test.

## 3. Results

**Figure 1:**
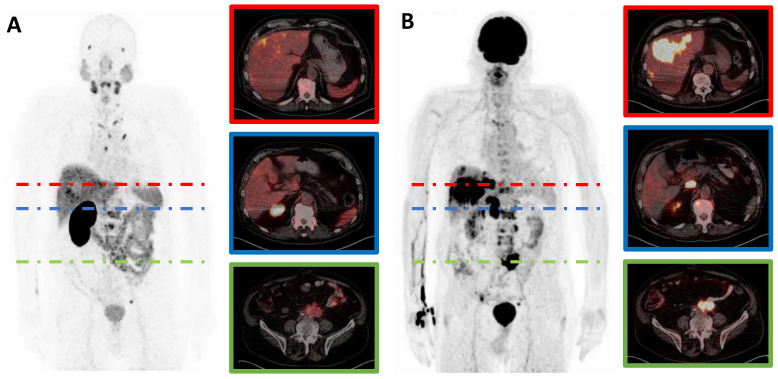
[^68^Ga]Ga-PSMA-11 (**A**) and [^18^F]FDG (**B**) PET/CT images of a representative mCRPC patient with hepatic (red) and lymphonodular (blue and green) mismatch lesions. Note: mCRPC, metastatic castration-resistant prostate cancer.

Out of *n* = 29 mCRPC patients included in this retrospective study, 17/29 (59%) patients showed at least one mismatch metastasis in combined [^18^F]FDG and [^68^Ga]Ga-PSMA-11 PET imaging (after a median of three cycles of PSMA-RLT), whereas 12/29 (41%) patients did not show any mismatch findings (after a median of five cycles of PSMA-RLT). In a subgroup of patients with mismatch, the median number of mismatch-metastases per patient was 8 (range 2–36). These mismatch metastases were found in bone (84 lesions in 11/17 patients); in lymph nodes (27 lesions in 9/17 patients), in the liver (69 lesions in 9/17 patients) and in the lung (6 lesions in 3/17 patients). Median MTV_m_ was 74.4 mL (range: 3.3–354.6 mL) per patient. Median TLG_m_ was 607.8 mL × SUV (range 13.0–4974.1 mL × SUV) per patient. Median SUV_max_ of the most intense mismatch lesion on [^18^F]FDG PET/CT was 16.8 (range: 7.0–95.2). Figure 1 shows a representative example of an mCRPC patient with multiple mismatch findings. No correlation was found between PSA and either SUV_max_ (*r* = −0.25, *p* = 0.926), MTV_m_ (*r* = 0.098, *p* = 0.708), or TLG_m_ (*r* = 0.235, *p* = 0.364).

**Figure 2:**
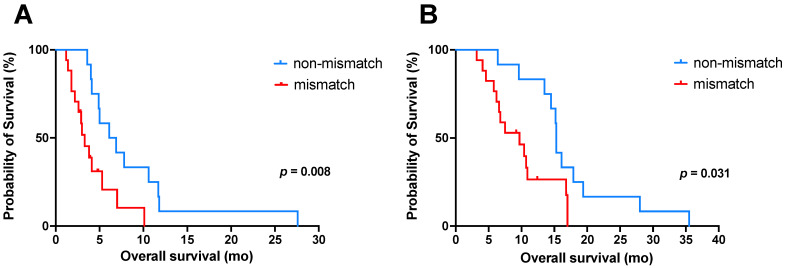
Kaplan–Meier curves for OS (**A**) from time point of combined [^18^F]FDG and [^68^Ga]Ga-PSMA-11 PET imaging and (**B**) from the start of [^177^Lu]Lu-PSMA-617 radioligand therapy, both stratified by mismatch (red) and non-mismatch (blue). Note: OS, overall survival.

In total, 13/17 patients with mismatch findings continued the PSMA-RLT with a median of 1 cycle (range 1–5 cycles). Of these, 4/13 patients received additional [^225^Ac]Ac-PSMA-617/[^177^Lu]Lu-PSMA-617 tandem RLT, and in 2/13 patients, co-medication with enzalutamide as potential radiosensitizer was restarted. The patient who received further five cycles of PSMA-RLT had only two small bone mismatch findings. Follow-up imaging with [^18^F]FDG PET/CT was only available in four patients with mismatch lesions. The known mismatch metastases were all progressive on follow-up imaging. All patients without mismatch (*n* = 12) continued the PSMA-RLT with a median of 2 cycles (range 1–4 cycles). Out of these, 6/12 patients received additional [^225^Ac]Ac-PSMA-617/[^177^Lu]Lu-PSMA-617 tandem RLT, and in 4/12 patients, co-medication with enzalutamide was restarted. By the end of this study, 26/29 patients (90%) had died. All deaths were mCRPC-related. No treatment-related death was observed. From the time point of PET imaging, the median OS (95% confidence interval (CI) of all patients was 4.1 mo (95%CI: 3.0–5.3 mo); patients with mismatch findings showed a significantly (*p* = 0.008) shorter OS of 3.3 mo (95%CI: 2.1–4.5 mo) than patients without mismatch with an OS of 6.1 mo (95%CI 2.9–9.3 mo). From the start of RLT, the median OS of all patients was 13.5 mo (95%CI: 8.5–18.6 mo). Again, patients with mismatch findings revealed a significantly (*p* = 0.031) shorter OS than patients without mismatch. The median OS of patients with mismatch metastases was 9.7 mo (95%CI: 5.1–14.2 mo), whereas the median OS of patients without was 15.3 mo (95%CI: 15.1–15.5 mo). The corresponding Kaplan–Meier curves are illustrated in Figure 2.

**Figure 3:**
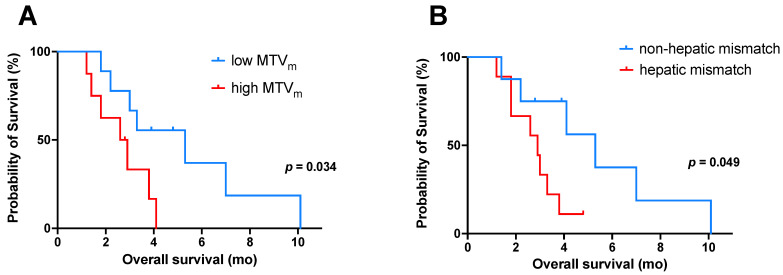
Kaplan–Meier curves for OS (from the time point of combined [^18^F]FDG and [^68^Ga]Ga-PSMA-11 PET imaging) in patients with mismatch findings (*n* = 17) stratified by (**A**) low/high MTV_m_ (cut-off value: 74.4 mL), and (**B**) presence or absence of hepatic mismatch lesions. Note: OS, overall survival; MTVm, metabolic tumor volume of all mismatch lesions.

To analyze prognostic factors, further subgroup analyses of patients with mismatch finding were performed regarding the [^18^F]FDG PET-derived parameters MTV_m_, TLG_m_, SUV_max_, and presence of hepatic mismatch lesions. In these subgroup analyses, patients with a high MTV_m_ (cut-off value 74.4 mL) revealed a significantly (*p* = 0.034) shorter OS (from time point of combined [^18^F]FDG and [^68^Ga]Ga-PSMA-11 PET imaging) than patients with low MTV_m_. The median OS was 2.6 mo (95% CI 1.3–3.9 mo) in patients with high MTV_m_ and 5.3 mo (95% CI 1.4–9.2 mo) in patients with low MTV_m_ (Figure 3A). There was also a trend to shorter survival in patients with mismatch metastases showing high SUV_max_ (cut-off value 16.8) or high TLG_m_ (cut off value 607.8 mL × SUV) in comparison to patients with low SUV_max_ or low TLG_m_, respectively (Figure S1); however, these differences in survival were not statistically significant (*p* = 0.070, *p* = 0.097). In addition, using [^18^F]FDG PET-derived parameters as metric variables, univariate Cox regression analysis of this subgroup also revealed a significant association between OS and MTV_m_ (hazard ratio 1.01; *p* = 0.006), whereas the other two [^18^F]FDG PET-derived parameters (SUV_max_ and TLG_m_) were not associated with OS (*p* = 0.484 and *p* = 0.242, respectively). Multivariable analysis was not performed due to the sample size being too small. Furthermore, patients with hepatic mismatch lesions showed a significantly (*p* = 0.049) shorter OS than patients without. The median OS was 2.9 mo (95% CI: 2.0–3.8 mo) in patients with hepatic mismatch lesions and 5.3 mo (95% CI: 2.8–7.8 mo) in patients without (Figure 3B). A further survival analysis (OS) for patients with mismatch lesions from the start of PSMA-RLT showed no significant difference in OS regarding the tested parameter (Figure S2).

## 4. Discussion

This study demonstrated the importance of combined [^18^F]FDG and [^68^Ga]Ga-PSMA-11 PET imaging in mCRPC patients whose disease worsened under ongoing [^177^Lu]Lu-PSMA-617-RLT by identifying patients with metastases showing intense glucose metabolism, but missing or low PSMA expression (mismatch metastases). These findings are associated with poor outcome.

The use of [^18^F]FDG PET/CT in addition to PSMA-targeted PET/CT to identify different phenotypes of disease (mismatch vs. non-mismatch) during [^177^Lu]Lu-PSMA-617 RLT is a novel approach. In our cohort of *n* = 29 mCRPC patients, who showed deterioration of disease state, the presence of discordant [^18^F]FDG-avid lesions without sufficient PSMA expression was associated with poor outcome. We observed a significantly shorter OS in patients with mismatch metastases than in patients without (median OS from the time of combined PET imaging: 3.3 mo vs. 6.1 mo; from the start of PSMA-RLT: 9.7 mo vs. 15.3 mo, respectively). It can be speculated that in the advanced stage of prostate cancer, tumor cells lose their PSMA expression as a mechanism of increased resistance induced by selective treatment pressure and become more aggressive. The information about the presence of such findings may be essential for further treatment management, particularly with regard to the potential benefit of continued PSMA-RLT, either in conventional or adjusted form. For example, in case of absence of mismatch findings, intensified PSMA-RLT by the use of [^225^Ac]Ac-PSMA-617 either as monotherapy or the [^225^Ac]Ac-PSMA-617/[^177^Lu]Lu-PSMA-617 tandem therapy approach have shown promising results regarding response and survival in patients with inadequately response to [^177^Lu]Lu-PSMA-617 monotherapy [25,26,27]. Moreover, upregulation of PSMA expression using enzalutamide as a potential radiosensitizer [28] may be an option to enhance therapy outcome. In contrast, development of mismatch metastases may require adding or switching to another treatment modality (e.g., chemotherapy, PARP inhibitors, or radiotherapy).

Our results were in line with those reported in previous studies by Thang et al. and Michalski et al., who also showed a negative prognostic factor of presence of mismatch metastases [29,30]. Thang et al. conducted dual-tracer imaging before enrollment in [^177^Lu]Lu-PSMA-617 RLT. These patients were excluded from PSMA-RLT and received the standard of care, exhibiting a median OS of 2.5 mo [29]. Similar to Thang et al., Michalski et al. screened patients for mismatch metastases prior to the start of PSMA-RLT; however, in the latter study, patients were treated with [^177^Lu]Lu-PSMA-617 if the majority of metastases was PSMA-positive and no other therapeutic options were available (median OS 6.0 mo) [30]. A direct comparison to our cohort of patients seems therefore inappropriate. Nevertheless, all studies indicated a poor outcome in case of mismatch findings.

To our knowledge, this is the first study that dealt with quantitative [^18^F]FDG PET-derived parameters in mismatch metastases and investigated their prognostic implication during [^177^Lu]Lu-PSMA-617 RLT. In the subgroup analysis of the patients with mismatch (*n* = 17), the MTV of all mismatch lesions (MTV_m_) could be identified as a negative prognostic factor for OS. Patients with high MTV_m_ showed significantly (*p* = 0.034) shorter OS than those with low MTV_m_ (median OS: 2.6 mo vs. 5.3 mo, respectively). MTV_m_ reflects the metabolically active tumor burden without or with only low PSMA expression, which may not be adequately treated by PSMA-RLT. Those lesions seemed to be transformed to a clearly more aggressive phenotype (i.e., dedifferentiation or transformation to neuroendocrine variant), which is generally associated with a very poor prognosis [31]. Regarding the other [^18^F]FDG PET-derived parameters (SUV_max_ and TLG_m_), there was a trend to shorter survival in patients with mismatch metastases showing high SUV_max_ and/or high TLG_m_; however, this difference in survival was not statistically significant. There are only a few studies of [^18^F]FDG-PET/CT and survival in advanced metastatic prostate cancer; however, all investigated the role of this complementary imaging method at baseline; i.e., before initiation of a new treatment [32,33,34]. Ferdinandus et al. [32] demonstrated that [^18^F]FDG PET at baseline is prognostic for survival in *n* = 50 mCRPC patients treated with [^177^Lu]Lu-PSMA-617 in a prospective phase II LuPSMA trial [12,35]. Patients with low volumes of [^18^F]FDG-avid disease had a longer OS than other patients. However, regarding the inclusion criteria of the LuPSMA trial, patients with mismatch lesions at baseline were excluded. Jadvar et al. reported the prognostic value of summed SUV_max_ on [^18^F]FDG PET in *n* = 87 mCRPC patients before receiving chemotherapy [33]. A high summed SUV_max_ value was associated with lower survival. Recently, Wibmer et al. demonstrated the value of whole-body tumor burden derived from baseline [^18^F]FDG PET/CT in patients with prostate cancer before first-line treatment with abiraterone or enzalutamide [34]. They found significant associations between OS probability and [^18^F]FDG PET-derived metrics (SUV_max_, number of [^18^F]FDG-avid metastases, whole-body MTV, and TLG). However, of these imaging parameters, only whole-body TLG was independently associated with OS probability. Although the constellations are different, all studies indicated the prognostic value of [^18^F]FDG PET-derived parameters in patients with prostate cancer.

Another notable finding of our study was that patients with mismatch metastases in the liver had significantly shorter OS than patients with mismatch metastases outside of the liver (median OS: 2.9 mo vs. 5.3 mo, *p* = 0.049). The presence of hepatic mismatch metastases can therefore be considered as an additional negative prognostic factor for OS in the subgroup of mismatch patients. It is known that mCRPC patients with liver metastases, independent of additional metastases in other tissues, had shorter survival than those without [36]. In a previous study, we demonstrated that the liver metastases could be frequently controlled by [^177^Lu]Lu-PSMA-617 RLT, resulting in long hepatic progression-free survival and significantly improved OS [37]. To date, little is known about the impact of hepatic mismatch findings in patients with mCRPC. In such scenarios with liver-dominant mismatch findings, locoregional therapy such as radioembolization may provide an effective treatment option in combination with systemic PSMA-RLT [38].

### Limitations

The results reported herein should be considered in the light of some limitations. First of all, the study was a single-center experience, which suffered from the limited number of patients and its retrospective nature. In addition, a quantitative SUV cut-off value for mismatch finding is still to be defined, and studies on this subject are recommended. Because combined [^18^F]FDG and [^68^Ga]Ga-PSMA-11 PET imaging was performed only when disease worsened and not routinely at fixed intervals, the actual time of development of mismatch metastases could not be accurately determined. Therefore, performing combined imaging before initiation of PSMA-RLT needs to be investigated in further studies to evaluate the impact of mismatch lesions on outcome (response and survival outcomes). Another limitation of this study was that, in view of the low specificity of [^18^F]FDG avidity, no other diagnostic methods (such as biopsy or [^68^Ga]Ga-DOTA-TATE PET/CT) were performed to investigate the nature of the mismatch lesions. Further specifications of mismatch findings (such as histopathological examination) should be included in future prospective studies. Lastly, more than half of the patients with mismatch lesions had liver metastases at baseline (compared to only one-sixth of the patients without mismatch findings), which may have had an additional negative impact on survival in this group.

## 5. Conclusions

In mCRPC patients with worsening disease during PSMA-RLT, combined [^18^F]FDG and [^68^Ga]Ga-PSMA-11 PET imaging is essential to identify patients with mismatch findings. These are associated with significantly poor outcomes, especially in case of high tumor volume or location in the liver. Consequently, in case of mismatch findings, addition or switch to another treatment modality is required. Further studies, ideally in prospective settings with larger patient cohorts, are needed to confirm and extend our results.

## Figures and Tables

**Table 1:** Patient characteristics.

Characteristics	All	Mismatch	Non-Mismatch
**Number of patients**	29	17	12
**Age**, median (range) in years	70 (51–88)	69 (51–75)	79 (57–88)
**PSA**, median (range) in ng/dL	205 (37–4742)	154 (37–1360)	282 (82–4742)
**Time from initial diagnosis**, *n* (%)			
≤2 years	8 (28%)	8 (47%)	0
>2–≤5 years	11 (38%)	6 (35%)	5 (42%)
>5 years	10 (34%)	3 (18%)	7 (58%)
**Prior therapy**, *n* (%)			
Prostatectomy	14 (48%)	6 (35%)	8 (67%)
Radiation	14 (48%)	7 (41%)	7 (58%)
ADT	29 (100%)	17 (100%)	12 (100%)
Enzalutamide	25 (86%)	14 (82%)	11 (92%)
Abiraterone	24 (83%)	12 (71%)	12 (100%)
Docetaxel	20 (69%)	13 (76%)	7 (58%)
Cabazitaxel	13 (45%)	9 (53%)	4 (33%)
^223^Ra-dichloride	4 (14%)	3 (18%)	1 (8%)
**ECOG PS**, *n* (%)			
0	8 (28%)	5 (29%)	3 (25%)
1	17 (59%)	9 (53%)	8 (67%)
2	3 (10%)	3 (18%)	0 (0%)
3	1 (3%)	0 (0%)	1 (8%)
**Site of metastases**, *n* (%)			
Bone	28 (97%)	16 (94%)	12 (100%)
Lymph node	20 (69%)	12 (71%)	8 (67%)
Liver	11 (38%)	9 (53%)	2 (17%)
Lung	5 (17%)	3 (18%)	2 (17%)

Note: PSA, prostate-specific antigen; ADT, androgen deprivation therapy; ECOG PS, Eastern Cooperative Oncology Group performance status. Categories in bold letter.

## Data Availability

The datasets used and analyzed during the current study are available from the corresponding author upon reasonable request.

## Supplementary Materials

The following are available online at https://www.mdpi.com/article/10.3390/cancers13164134/s1, Figure S1. Kaplan-Meier curves for OS (from the time point of combined [^18^F]FDG and [^68^Ga]Ga-PSMA-11 PET imaging) in patients with mismatch findings (*n* = 17) stratified by (A) low/high SUV_max_ (cut-off value: 16.8): median OS 3.8 mo (95% CI 0.4–7.2 mo.)/ 2.9 mo (95% CI 2.2–3.6 mo.), (B) low/high TLG_m_ (cut-off value: 607.8 mL x SUV): median OS 3.3 mo (95% CI 0–7.0 mo)/2.9 mo (95% CI 2.1–3.6 mo), Figure S2. Kaplan-Meier curves for OS (from the start of [^177^Lu]Lu-PSMA-617 radioligand therapy) in patients with mismatch findings (*n* = 17) stratified by (A) low/high MTV_m_ (cut-off value: 74.4 mL), (B) low/high SUV_max_ (cut-off value: 16.8), (C) low/high TLG_m_ (cut-off value: 607.8 mL x SUV), (D) presence or absence of hepatic mismatch lesions.
