# Supplementary material for: Value of Combined PET Imaging with [18F]FDG and [68Ga]Ga-PSMA-11 in mCRPC Patients with Worsening Disease during [177Lu]Lu-PSMA-617 RLT
Source: Cancers (Basel). 2021 Aug 17;13(16):4134. doi: 10.3390/cancers13164134 (PMC8391978; doi:10.3390/cancers13164134)
Supplement: Supplementary file 1 [file cancers-13-04134-s001.zip › cancers-1325343-supplementary.pdf]

cancers

MDPI

Supplementary Materials: Value of Combined PET Imaging with [18F]FDG and [68Ga]Ga-PSMA-11 in mCRPC Patients with Worsening Disease during [177Lu]Lu-PSMA-617 RLT

Fadi Khreish, Kalle Ribbat, Mark Bartholomä, Stephan Maus, Tobias Stemler, Ina Hierlmeier, Johannes Linxweiler, Mathias Schreckenberger, Samer Ezziddin and Florian Rosar

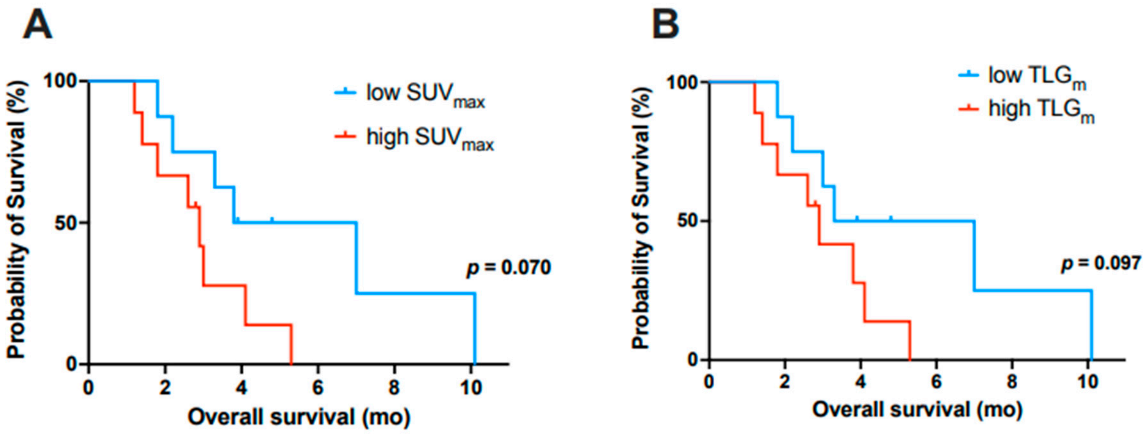

Figure S1. Kaplan-Meier curves for OS (from the time point of combined [18F]FDG and [68Ga]Ga-PSMA-11 PET imaging) in patients with mismatch findings (n = 17) stratified by (**A**) low/high SUV_{max} (cut-off value: 16.8): median OS 3.8 mo (95% CI 0.4–7.2 mo.)/ 2.9 mo (95% CI 2.2–3.6 mo.), (**B**) low/high TLG_m (cut-off value: 607.8 mL x SUV): median OS 3.3 mo (95% CI 0–7.0 mo)/2.9 mo (95% CI 2.1–3.6 mo). OS: overall survival; SUV_{max}: maximal standardized uptake value of the mismatch lesion with the highest [18F]FDG uptake; CI: confidence interval; TLG_m: total lesion glycolysis of all mismatch lesions.

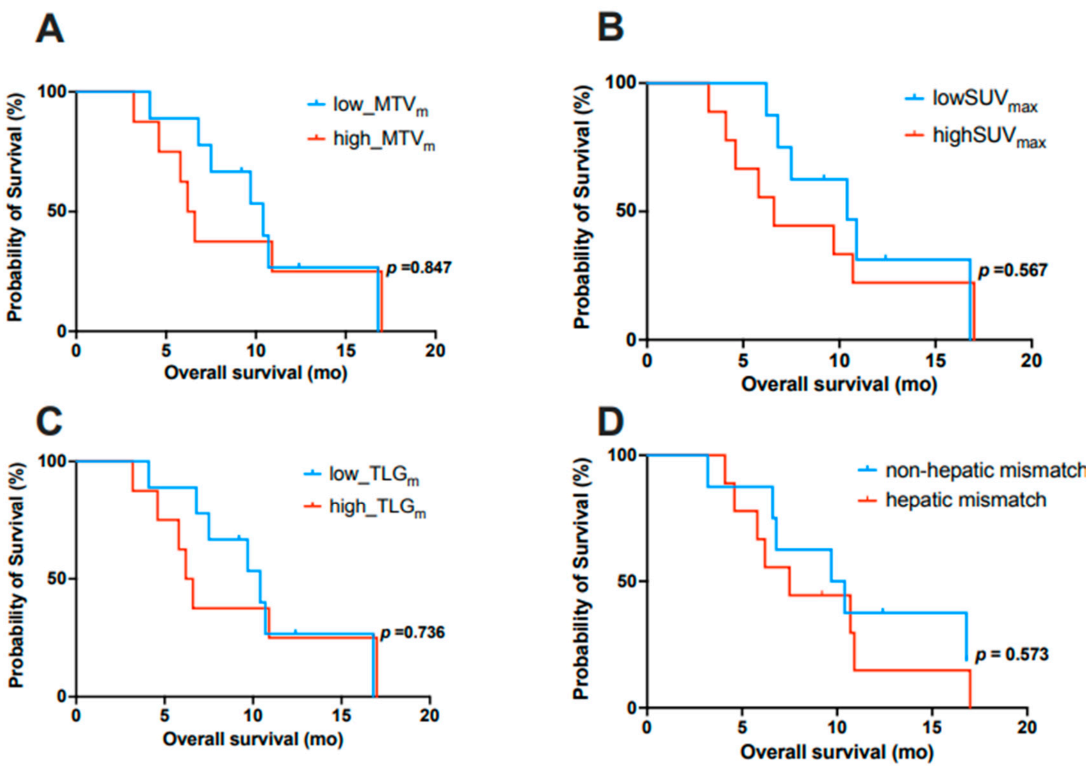

Figure S2. Kaplan-Meier curves for OS (from the start of [^{177}Lu]Lu-PSMA-617 radioligand therapy) in patients with mismatch findings (n = 17) stratified by (**A**) low/high MTV_m (cut-off value: 74.4 mL), (**B**) low/high SUV_{max} (cut-off value: 16.8), (**C**) low/high TLG_m (cut-off value: 607.8 mL x SUV), (**D**) presence or absence of hepatic mismatch lesions. OS: overall survival; MTV_m: metabolic tumor volume of all mismatch lesions; SUV_{max}: maximal standardized uptake value of the mismatch lesion with the highest [^{18}F]FDG uptake; TLG_m: total lesion glycolysis of all mismatch lesions.
